# Enhanced Antiproliferative and Apoptotic Response of HT-29 Adenocarcinoma Cells to Combination of Photoactivated Hypericin and Farnesyltransferase Inhibitor Manumycin A

**DOI:** 10.3390/ijms12128388

**Published:** 2011-11-29

**Authors:** Veronika Sačková, Lucia Kuliková, Martin Kello, Ivana Uhrinová, Peter Fedoročko

**Affiliations:** Institute of Biology and Ecology, Faculty of Sciences, P. J. Šafárik University, Moyzesova 11, Košice 041 65, Slovakia; E-Mails: veronika.sackova@upjs.sk (V.S.); lucia_kulikova@yahoo.com (L.K.); kellomartin@yahoo.com (M.K.); ivana_uhrinova@yahoo.com (I.U.)

**Keywords:** hypericin, manumycin, apoptosis, farnesyltransferase

## Abstract

Several photodynamically-active substances and farnesyltransferase inhibitors are currently being investigated as promising anticancer drugs. In this study, the combined effect of hypericin (the photodynamically-active pigment from *Hypericum perforatum*) and selective farnesyltransferase inhibitor manumycin (manumycin A; the selective farnesyltransferase inhibitor from *Streptomyces parvulus*) on HT-29 adenocarcinoma cells was examined. We found that the combination treatment of cells with photoactivated hypericin and manumycin resulted in enhanced antiproliferative and apoptotic response compared to the effect of single treatments. This was associated with increased suppression of clonogenic growth, S phase cell cycle arrest, elevated caspase-3/7 activity and time-dependent total cleavage of procaspase-3 and lamin B, cleavage of p21Bax into p18Bax and massive PARP cleavage. Moreover, we found that the apoptosis-inducing factor is implicated in signaling events triggered by photoactivated hypericin. Our results showed the relocalization of apoptosis-inducing factor (AIF) to the nuclei after hypericin treatment. In addition, we discovered that not only manumycin but also photoactivated hypericin induced the reduction of total Ras protein level. Manumycin decreased the amount of farnesylated Ras, and the combination treatment decreased the amount of both farnesylated and non-farnesylated Ras protein more dramatically. The present findings indicate that the inhibition of Ras processing may be the determining factor for enhancing the antiproliferative and apoptotic effects of combination treatment on HT-29 cells.

## 1. Introduction

Hypericin is a photodynamic pigment first isolated from the plant *Hypericum perforatum* L., commonly known as St. John’s Wort [1]. The term “photodynamic” means that hypericin as a component of photodynamic therapy becomes activated by visible light at the proper wavelength (570–650 nm) in the presence of molecular oxygen. The subsequent generation of singlet oxygen and superoxide anions, led to oxidative damage and destruction of cancer cells [2,3].

Treatment with hypericin-mediated photodynamic therapy is under investigation for several cancer and non-cancerous diseases. In an effort to improve the treatment effectiveness, various combination approaches have been investigated. Our previous studies showed increased apoptosis of various cancer cell lines when hypericin-mediated photodynamic therapy was combined with 5-LOX inhibitor [4], P450 monooxygenase inhibitor [5], genistein [6] and polyunsaturated fatty acids [7]. The effectiveness of hypericin-mediated photodynamic therapy was improved by the use of PD169316, a p38α MAPK inhibitor in human cervix carcinoma cells and human bladder cancer cells [8] and by diazepam in glioma cells [9]. Hypericin was able to enhance radiosensitivity in human malignant glioma cells and human renal carcinoma cells [10,11] and the antiglioma effects of temozolomide by inducing apoptosis both *in vitro* and *in vivo* [12]. The combination of hypericin-mediated photodynamic therapy with hyperthermia enhanced RIF-1 tumor cell killing by triggering apoptosis [13]. Bhuvaneswari *et al.* documented increased apoptosis associated with bladder tumor inhibition using the combination of hypericin-mediated photodynamic therapy with angiogenesis inhibitors [14].

Manumycin A (UCF1-C) is a natural product from *Streptomyces parvulus* that acts as a potent and selective Ras farnesyltransferase inhibitor [15]. The enzyme farnesyltransferase modifies Ras and other proteins with the farnesyl isoprenoid lipid that is required for their correct cellular localization and biological activity [16].

Recently, the anti-neoplastic activity of manumycin has been demonstrated in various experimental systems. Manumycin-induced apoptosis of human pancreatic cancer cells [17], anaplastic thyroid cancer cells [18,19], human colon tumor cells [20], human hepatocellular carcinoma HepG2 cells [21], medulloblastoma cells [22,23], leukemic U937 and HL-60 cells [24], lymphoid tumor and myeloma cell lines [25,26].

Several studies have demonstrated the enhanced cytotoxic or apoptotic effects on various cancer cell lines as a consequence of the combination of manumycin and paclitaxel [18], methoxyamine [27] and HSP inhibitor quercetin [28]. The combination of manumycin and paclitaxel and the triple-drug combination of manumycin, paclitaxel and minocycline were effective also *in vivo* against anaplastic thyroid carcinoma [29,30].

In this work, the effective combination of photodynamically-active drug and selective farnesyltransferase inhibitor was investigated for the first time. Besides an enhanced antiproliferative and apoptotic response of HT-29 cells to combination treatment with photoactivated hypericin and manumycin, we also discovered new players in the signaling machinery triggered by photoactivated hypericin, namely an apoptosis-inducing factor (AIF) and Ras.

Our results indicate the possibility of new effective combination of two natural products, the photodynamically-active drug and farnesyltransferase inhibitor, as a new modality approach for anticancer therapies in the future.

## 2. Results and Discussion

**Figure 1:**
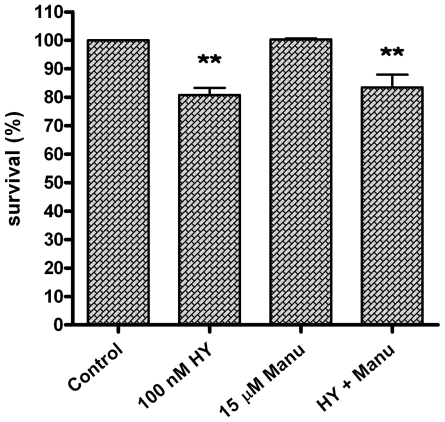
MTT assay. Cells were treated with drugs (HY—hypericin, Manu—manumycin) as indicated or left untreated (Control), and MTT assay was performed 24 h after hypericin photoactivation. All data are expressed as mean ± SEM from three independent experiments (^**^ *p* < 0.01 compared to control).

In this study, colon adenocarcinoma cells HT-29 were exposed to combination treatment with photoactivated hypericin and Ras farnesyltransferase inhibitor manumycin. Hypericin was used at a concentration of 100 nM, which induces apoptosis and G2 phase arrest of HT-29 cells under defined conditions of the photodynamic protocol, as we demonstrated in our previous study [31]. Manumycin at a concentration of 15 μM was added to cells 1 h before hypericin photoactivation. As the results of MTT assay showed, this concentration was not cytotoxic for the cells and it did not modify the effect of hypericin as determined by MTT values of the combination treatment (Figure 1).

**Figure 2:**
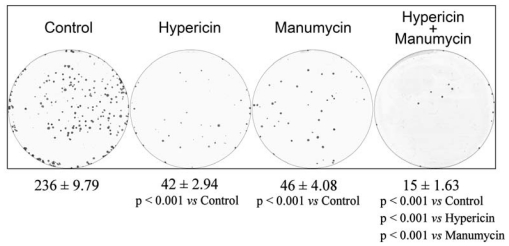
Colony-forming assay. Cells were treated with drugs (HY—hypericin, Manu—Manumycin) as indicated or left untreated (Control). After the treatments, equal numbers of viable cells (500/well) were seeded into 6-well plates containing RPMI-1640 and FBS (10%) and allowed to grow under standard conditions for 10 days. Colonies were stained using methyl blue solution. All data are expressed as mean ± SEM from three independent experiments.

In contrast to MTT, the cells showed decreased colony-forming capacity after manumycin treatment and also after hypericin treatment compared to untreated control. The combination treatment led to enhanced inhibition of colony formation compared to treatment with hypericin or manumycin alone (Figure 2). The different mechanisms involved in each assay probably explain the discrepancy between the MTT and clonogenic assay results, *i.e.*, short-term cytotoxicity in the MTT assay and long-term proliferation and clonogenic survival in the clonogenic assay [32].

**Figure 3:**
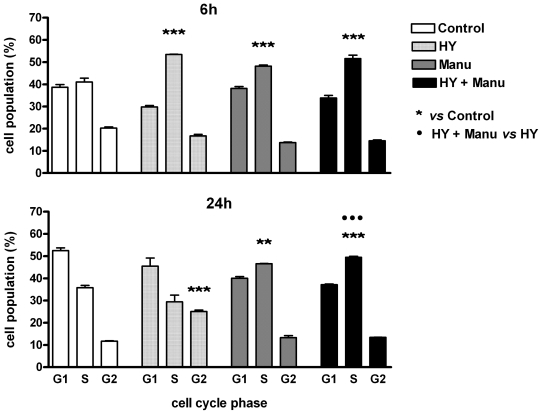
Cell cycle distribution. Cells were treated with drugs (HY—hypericin, Manu—Manumycin) as indicated or left untreated (Control), and cell cycle analysis using flow cytometry was performed at 6 and 24 h after hypericin photoactivation. All data are expressed as mean ± SEM from three independent experiments (^**^ *p* < 0.01, ^***^ *p* < 0.001; ^•••^ *p* < 0.001).

To determine whether cell cycle arrest contributed to the decreased cell proliferation observed after combination therapy, cell cycle analysis using flow cytometry was performed. The data on cell cycle distribution (Figure 3) revealed that cells treated with the drug combination were accumulated in the S phase (51.59% *versus* 41.03% in control) as well as cells treated with manumycin (48.17% *versus* 41.03% in control) or hypericin alone (53.46% *versus* 41.03% in control) at 6 h. Cells treated with manumycin alone (46.65% *versus* 35.79% in control) and with combination (49.47% *versus* 35.79% in control) also remain accumulated in the S phase after longer incubation period (24 h). In cells treated with hypericin, the early S phase arrest disappeared and cells were arrested in the G2 phase of cell cycle at 24 h (25.05% *versus* 11.71% in control). This means that hypericin-treated cells passed through the S phase and were subsequently blocked in G2 phase arrest. Although hypericin alone led to G2 arrest, the cells treated with the combination did not arrest in G2 but accumulated in the S phase. These results demonstrate that accumulation of cells in the S phase after the combination treatment is significant compared to hypericin alone, but the cell cycle distribution is the same as the pattern induced by manumycin alone. Moreover, the disappearance of both S and G2 arrests induced by hypericin at the later time points indicate that these processes are reversible. In contrast, the manumycin-caused S phase arrest was irreversible and sustained from early to later analysis time points.

Previous works of our research group have documented G2 phase arrest after hypericin treatment in HT-29 cells [31] and hypericin plus genistein treatment in MCF-7 cells [6], S phase arrest in hypericin-treated U937 cells [31] and S phase arrest also with accumulation of cells in the G0/G1 phase induced by combination of hypericin and LOX inhibitor MK-886 [4]. Increased subdiploid G0/G1 population of HL-60 cells was detected after hypericin plus acetazolamide treatment [33]. Vantieghem *et al.* reported G2/M phase-arrested HeLa cells following photodynamic therapy with hypericin [34].

Manumycin A had no effect on the cell cycle of medulloblastoma cells [22]. In contrast, HepG2 cells were accumulated in the G2/M of the cell cycle after treatment with manumycin in the study by Zhou *et al.* [21]. These reports indicate that different mechanisms for hypericin or manumycin induced cytotoxicity may be involved in different cancer cell types.

The results of cell cycle distribution are consistent with the growth inhibition detected by colony-forming assay, suggesting that the antiproliferative effect of drugs alone and in combination is associated with S phase arrest and apoptosis of cells. It has been reported that both arrest and death preclude outgrowth of colonies [35].

**Figure 4:**
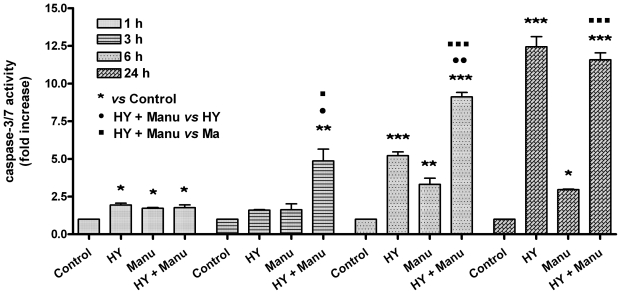
Caspase-3/7 activity. Cells were treated with drugs (HY—hypericin, Manu—Manumycin) as indicated or left untreated (Control), and caspase-3/7 activity was measured using Apo-One™ homogenous caspase-3/7 assay at various times after hypericin photoactivation. All data are expressed as mean ± SEM from three independent experiments as ratios to untreated control values (^*^ *p* < 0.05, ^**^ *p* < 0.01, ^***^ *p* < 0.001; ^•^ *p* < 0.05, ^••^ *p* < 0.01; ^▪^ *p* < 0.05, ^▪▪▪^ *p* < 0.001).

Each of the apoptotic signaling pathways converges to a common execution phase of apoptosis that requires proteolytic activation of caspases-3 and/or -7 from their inactive zymogens. Measurement of caspase-3/7 activity in a fluorometric assay revealed elevated enzyme activity after combination treatment (Figure 4). The results showed that combination treatment induced statistically significant time-dependent increase in caspase-3/7 activation at 3 and 6 h time points compared to control and hypericin or manumycin alone. Treatment of cells with hypericin and manumycin alone elevated caspase-3/7 activity as early as 1 h compared to control, and it remained elevated above control at 6 h and 24 h. Caspase-3/7 activation was completely eliminated by pan-caspase inhibitor Z-VAD-FMK.

**Figure 5:**
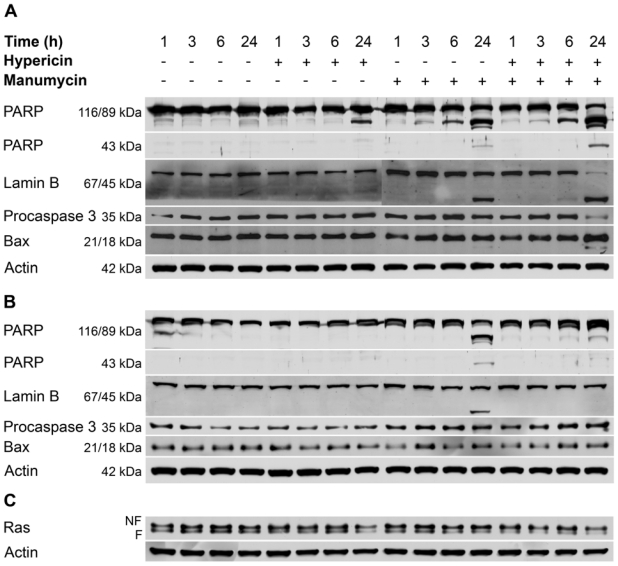
Western blot analysis of apoptosis-related proteins (**A**,**B**) and total Ras (**C**) in whole cell extracts prepared at indicated time periods after the various treatments. Cells were incubated with the general caspase inhibitor Z-VAD-FMK at a concentration of 50 μM for 2 h prior to hypericin photoactivation (**B**). The immunoblots shown here represent three independent experiments with similar results. β-actin (Actin) was employed as the loading control. (NF—non-farnesylated, F—farnesylated).

To determine whether the decreased proliferation activity of cells and increased caspase activity after combination treatment were associated with the induction of apoptosis, the apoptotic proteins were analyzed using western blotting (Figure 5A). As the results show, the long incubation period (24 h) revealed the enhanced effect of hypericin plus manumycin treatment on the analyzed proteins compared to single treatments.

Manumycin alone or manumycin plus hypericin treatment caused more rapid proteolytic cleavage of PARP associated with accumulation of an 89-kDa fragment than hypericin alone. The 89-kDa PARP fragment was detected as early as 6 h after manumycin and combination treatment. Most of the 116-kDa PARP was degraded at 24 h in cells treated with hypericin plus manumycin. At that time of analysis, a massive 89-kDa PARP fragment occurred simultaneously with complete cleavage of the 67-kDa lamin B, which generated a fragment of 45 kDa, total processing of procaspase-3 (35-kDa) and appearance of a p18 fragment of Bax in cells treated with the combination of drugs. The cleavage fragment of Bax was weakly detected also in manumycin-treated cells. Interestingly, in cells treated with manumycin and hypericin plus manumycin, another fragment of PARP corresponding to ~43 kDa was observed along with the usual ~89 kDa.

The cleavage of full-length p21 Bax into proapoptotic p18 Bax is described in several studies [36–38]. According to published data, cleavage of full-length Bax into proapoptotic p18 Bax is mediated by calpain [39–41] and results in acceleration of apoptotic cell death [42,43].

To determine whether caspases were involved in cleavage of proteins, cells were pretreated with the broad-spectrum caspase inhibitor Z-VAD*-*FMK. As shown in Figure 5B, pre-treatment of cells with Z-VAD-FMK completely blocked hypericin-induced PARP cleavage into the ~89 kDa fragment. In manumycin-treated cells PARP cleavage was prevented only at the 3 and 6 h analyses, but the inhibitor had no effect on manumycin-induced PARP and lamin B cleavage at the 24 h analysis. Cleavage of PARP into the smaller ~43 kDa fragment was blocked by general pan-caspase inhibitor in cells treated with the combination of drugs, but not in manumycin-treated cells. This is the first evidence about caspase-independent PARP and lamin B cleavage alike in manumycin-induced caspase-dependent apoptosis.

Proteolytic cleavage of 116-kDa polypeptide PARP to its characteristic 89-kDa fragment is known as a typical marker of apoptosis. Although this cleavage is largely mediated by a number of caspases, recent studies have demonstrated caspase-independent PARP cleavage in TGF-β-induced caspase-dependent apoptosis of AML-12 cells and in MBP-1-induced lung cancer cell death [44,45]. In the study by Bhaskara *et al.* [46] apoptotic cell death led predominantly to the appearance of the larger ~89 kDa fragment and smaller ~24 kDa fragment, but also other fragments were formed indicating other forms of cell death. PARP-1 is also a preferred substrate for the proteolytic action of calpains and cathepsins that give rise to ~40 kDa fragments [47]. Since the cleavage of PARP into the smaller ~43 kDa fragment was blocked by general pan-caspase inhibitor in cells treated with the combination of drugs, we suppose that calpain is not involved in this process. In contrast, the ~43 kDa fragment of PARP detected in manumycin-treated cells was not eliminated by Z-VAD-FMK. This result may indicate the co-existence of various intermediate cell death pathways [46].

The lamins represent a key substrate for the proteases activated during apoptosis, whose degradation facilitates the nuclear events of apoptosis [48]. Proteolysis of lamin B has been observed in various cells undergoing apoptosis after different insults [49–53]. Lamin B was proteolyzed during apoptosis of HepG2 cells induced by manumycin [21]. The involvement of caspases in this process was not investigated. In our study, lamin B cleavage was not blocked by pan-caspase inhibitor in manumycin-treated cells, indicating that this process is not dependent on caspases and could also be achieved through proteases other than caspases. Zhivotovsky *et al.* demonstrated the involvement of at least two proteases in lamin cleavage [54]. Neither of them cleaved PARP, so they concluded that lamin and PARP cleavage during apoptosis are distinct processes mediated by different proteases.

However, PARP, lamin B and Bax cleavage together with procaspase 3 processing were completely blocked by pan-caspase inhibitor in cells treated with hypericin plus manumycin. These results along with inhibition of caspase-3/7 activity by Z-VAD-FMK indicate that the enhanced apoptotic response induced by combination treatment is realized via a caspase-dependent pathway.

To further elucidate the mechanism of enhanced apoptosis induced by combination treatments, the possibility of involvement of apoptosis-inducing factor (AIF) was examined. AIF is a pro-apoptotic mitochondrial intermembrane flavoprotein that is translocated to the nucleus and causes chromatin condensation in response to various stimuli including some photosensitizers [55–58].

**Figure 6:**
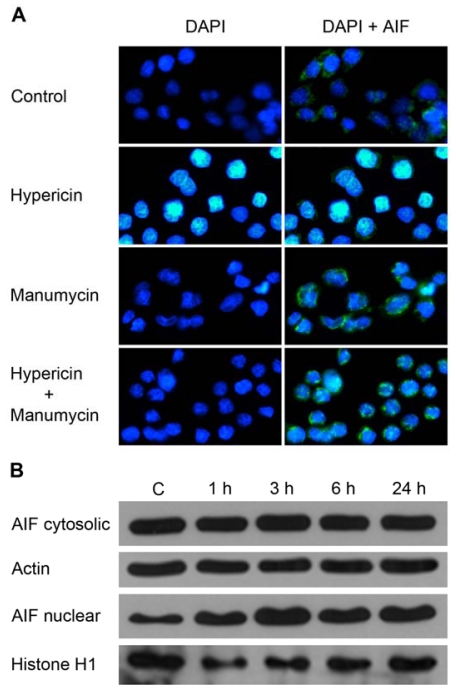
Relocalization of AIF to the nucleus. Cells were treated with drugs as indicated or left untreated (C; Control), and immunofluorescent staining protocol was performed at 3 h after hypericin photoactivation. Cells were stained with DAPI to visualize the nuclei (blue fluorescence). Cells were immunostained with an anti-AIF polyclonal antibody (green fluorescence) (**A**). Translocation of AIF was examined by western blotting of the cytosolic and nuclear fractions at various times after hypericin photoactivation. Equivalent protein loading was confirmed by appropriate loading markers (**B**).

In our study, apoptosis-inducing factor was translocated to the nucleus as an early response of cancer cells to hypericin treatment, but not to manumycin treatment. Immunofluorescence analysis revealed changes in nuclear morphology at 3 h following hypericin photoactivation. As demonstrated by DAPI staining, the typical condensed and fragmented apoptotic nuclei were detected. AIF staining revealed diffuse distribution in the cytosol and peripheral areas of condensed apoptotic nuclei of hypericin-treated cells, compared to granular pattern in the cytosol of untreated cells and also cells treated with manumycin or combination. This observation proves the translocation of AIF into the nuclei after hypericin photoactivation (Figure 6A), and it was also confirmed by Western blot analysis of the cytosolic and nuclear fraction. The results show that hypericin induces the relocalization of AIF to the nucleus. The level of AIF was elevated as early as 1 h after PDT and continued to increase for a 3 h period. The amount of AIF in the nuclear fraction remained elevated at 6 and 24 h compared to untreated cells (Figure 6B). These results indicate that caspases and AIF can function in parallel in hypericin but not in manumycin or combination-triggered apoptotic signaling events. To our knowledge, this is the first report about the involvement of AIF in hypericin photodynamic action.

Based on the fact that manumycin can acts as Ras inhibitor, it was of interest to examine whether hypericin can affect Ras as well. Determination of the level of total Ras by western blotting with anti pan-Ras antibody showed that hypericin reduced the level of both farnesylated (F) and non-farnesylated (NF) Ras at the 24 h analysis (Figure 5C). These results demonstrate for the first time the involvement of Ras in photoactivated hypericin signaling. To our knowledge, only one study exists that reports a decrease in Ras protein level triggered by Rose Bengal-mediated photodynamic treatment in HUVECs. In agreement with others [20–22,59], treatment with manumycin alone caused a decrease in farnesylated Ras protein. Furthermore, since HT-29 expresses a normal K-*ras* gene, our results support the findings of Di Paolo *et al.* that manumycin is able to suppress the proliferation of tumor cells with wild-type K-*ras* genes [20]. Changes in Ras level detected after combination treatment showed the time-dependent effect of both agents. The amount of both bands of Ras was reduced at all time points, but more pronounced decrease was noticed at the 24 h analysis.

Collectively, these findings suggest that the enhanced antiproliferative and apoptotic response of HT-29 adenocarcinoma cells after combination treatment is a consequence of strong decrease in total Ras protein, which is a common target of both photoactivated hypericin and manumycin action. Further investigations are required to determine the signaling events downstream of Ras.

## 3. Experimental Section

### 3.1. Cell Culture

The human colon adenocarcinoma cell line HT-29 (American Type Culture Collection, Rockville, MD, USA) was cultured in RPMI-1640 medium (Gibco, Grand Island, NY, USA) supplemented with 10% fetal bovinne serum (FBS; Invitrogen Corp., Carlsbad, CA, USA) and antibiotics (penicillin 100 U/mL, streptomycin 100 μg/mL and amphotericin 25 μg/mL; Invitrogen Corporation, Carlsbad, CA, USA). Cells were maintained at 37 °C in a humidified 5% CO_2_ atmosphere.

### 3.2. Reagents

Hypericin (4,5,7,4′,5′,7′-hexahydroxy-2,2′-dimethylnaphtodiantron, HPLC grade) was purchased from AppliChem GmbH (Darmstadt, Germany); MTT (3-(4,5-dimethylthiazol-2-yl)-2,5-diphenyl tetrazolium bromide), manumycin A from *Streptomyces parvulus* and Z-VAD-FMK (*N*-Benzyloxycarbonyl-Val-Ala-Asp(*O*-Me) fluoromethyl ketone) were obtained from Sigma-Aldrich Corporation (St. Louis, MO). Both hypericin and manumycin were prepared in DMSO as stock solutions stored at −20 °C (hypericin at 1 mM concentration; manumycin at 10 mM concentration) and then diluted to a working solution of final concentration (hypericin at 100 nM concentration; manumycin at 15 μM concentration). The final concentration of DMSO was less than 0.1% and did not influence the cytokinetic parameters.

### 3.3. Experimental Design

**Scheme 1:**
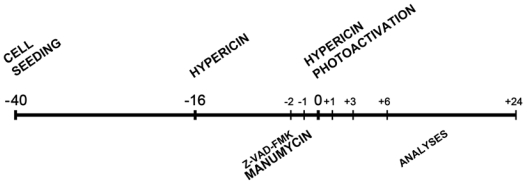
Experimental time schedule (h). Cells were seeded (−40 h) and cultivated for 24 h in a complete medium. Next, hypericin was added (−16 h) and photoactivated (0 h). Z-VAD-FMK and manumycin were added at 2 h and 1 h respectively before hypericin photoactivation. Analyses were performed at 1 h, 3 h, 6 h and 24 h after hypericin photoactivation.

HT-29 cells were seeded and cultivated for 24 h in a complete medium RPMI-1640 with 10% FBS. Next, cells were treated according to the time schedule (Scheme 1). The crucial point of the whole experimental design was the time of hypericin photoactivation (0 h).

### 3.4. Hypericin Treatment

Cells were incubated for 16 h with 100 nM hypericin in dark conditions before light activation with a single light dose (4.4 J·cm^−2^). The control group received a medium with serum without hypericin. Following hypericin photoactivation cells were incubated at 37 °C in dark conditions for the indicated times and separate analyses were performed.

### 3.5. Manumycin Treatment

Inhibitor manumycin (15 μM) was added to cells 1 h prior to hypericin photoactivation.

### 3.6. Pan-Caspase Inhibitor Treatment

Cells were incubated with the general caspase inhibitor Z-VAD-FMK at a concentration of 50 μM for 2 h prior to hypericin photoactivation.

### 3.7. Hypericin Photoactivation

Plates with the cells were placed on a plastic diffuser sheet above a set of nine L18W/30 lamps (Osram, Berlin, Germany) with maximum emission between 530 and 620 nm (the absorption peak of hypericin is 595 nm). At the surface of the diffuser the uniform fluence rate was 4.4 mW·cm^−2^·s^−1^ and the temperature did not exceed 37 °C.

### 3.8. MTT Assay

Cells were seeded in 96-well tissue culture plates (1 × 10^4^ cells/well). After the treatments, MTT (3-(4,5-dimethylthiazol-2-yl)-2,5-diphenyl tetrazolium bromide; Sigma-Aldrich Corporation) was added to cells at the analysis time point (24 h after hypericin photoactivation) and after 4 h incubation the metabolization of MTT was stopped and crystals of formazan were dissolved overnight with 10% SDS (final concentration 3.3%) (Serva, Heidelberg, Germany). The amount of dissolved formazan was quantified by absorbance measurements at 584 nm (FLUOstar Optima, BMG Labtechnologies GmbH, Offenburg, Germany) and expressed as a percentage of the dye extracted from untreated control cells ((OD value of treated cells/mean OD value of control cells) × 100%).

### 3.9. Colony-Forming Assay

To determine colony formation, cells were seeded into 6-well tissue culture plates (2 × 10^6^ cells/well) and treated according to the experimental design for 24 h. After this period, cells were trypsinized, collected and washed twice with fresh medium. Equal numbers of viable cells (500/well) were then seeded into 6-well tissue culture plates containing RPMI-1640 and FBS (10%) and allowed to grow under standard cultivation conditions until visible colonies were observed. Colonies were stained using methyl blue staining.

### 3.10. Cell Cycle Distribution Analysis

For DNA content, cells were harvested, washed with phosphate-buffered saline (PBS) and fixed with 70% ice-cold ethanol at −20 °C. Fixed cells were centrifuged, washed with PBS and stained with staining solution (5 mg/mL propidium iodide, 10 mg/mL RNase A and 10% Triton X-100 in PBS). Then samples were kept in dark conditions for 30 min and measured on a flow cytometer (FACS Calibur, Becton Dickinson, San Diego, CA). For each sample, 15,000 cells were evaluated and the sample flow rate during analysis did not exceed 200–300 cells per second. The data obtained were analyzed using the Cell Quest Pro software.

### 3.11. Caspase-3/7 Activity Assay

Caspase-3/7 activity was measured using Apo-ONE™ Homogeneous Caspase-3/7 Assay (Promega Corporation, Madison, WI, USA) based on the cleavage of the non-fluorescent caspase substrate Z-DEVD-R110 by caspase-3/7 to create fluorescent Rhodamine 110. Experiments were performed according to the protocol provided by the manufacturer. After the treatments according to the experimental design, the homogeneous caspase-3/7 reagent was added to the 2 × 10^4^ cells/sample. Following incubation at room temperature for 2 h, caspase-3/7 activity was estimated from the fluorescence measured using a fluorescence microplate reader (excitation at 499 nm, emission at 521 nm; FLUOstar Optima).

### 3.12. Western Blot Analysis

For preparation of whole cell lysates, 2 × 10^6^ cells/well were seeded into 6-well microplates and treated in accordance with the experimental protocol. Harvested cells were washed in cold PBS and lysed in lysis buffer (100 mM Tris-HCl, pH 7.4, 1% SDS, 10% glycerol) with protease inhibitor cocktail (P2714; Sigma-Aldrich Corporation) for 10 min on ice. The cell lysates were sonicated and centrifuged. Protein concentration was determined using detergent compatible protein assay (BioRad Laboratories Inc., Hercules, CA, USA). Equal amounts of proteins (30 μg) were diluted in sample buffer (1 M Tris-HCl, 10% SDS, 1% 2-mercaptoethanol, 1% bromphenol blue and 50% glycerol), separated in SDS-polyacrylamide gel and transferred onto PVDF membrane (Millipore, Bedford, MA, USA) in a transfer buffer containing 192 mM glycine, 25 mM Tris and 10% methanol. After membrane blocking (1 h at RT) in 5% non-fat milk in TBS-Tween (20 mM Tris-HCl, pH 7.6; 150 mM NaCl; 0.05% TWEEN 20, pH 7.4) the PVDF membrane blots were incubated with the primary (1 h at RT or overnight at 4 °C) and secondary antibodies (1 h at RT). Blots were developed with ECL western blotting substrate (Pierce Biotechnology, Inc., Rockford, IL, USA) and visualized on X-ray films (AGFA-gevaert N.V., Kontich, Belgium).

### 3.13. Cytosolic and Nuclear Fractionation

The extraction of nuclear and cytoplasmic protein fractions was performed with the Nuclear/Cytosol Fractionation Kit (BioVision Research Products, Mountain View, CA, USA) in line with the protocol provided by the manufacturer.

### 3.14. Antibodies

Primary antibodies included mouse monoclonal anti-caspase-3 (E-8, 1:1000), rabbit polyclonal anti-PARP (H-250, 1:1000), rabbit polyclonal anti-Bax (1:1000), goat polyclonal anti-Lamin B (M-20, 1:200) and goat polyclonal anti-histone H1 (N-16, 1:1000) purchased from Santa Cruz Biotechnology (Santa Cruz, CA, USA); rabbit polyclonal anti-AIF (1:1000) from Cell Signaling Technology, Inc. (Danvers, MA, USA), mouse monoclonal anti-β-actin (1:1000) from Sigma-Aldrich Corporation (St.Louis, MO, USA), and mouse monoclonal anti*-*Pan*-*Ras (Ab-3, 1:100) from Calbiochem (Merck KGaA, Darmstadt, Germany). Horseradish peroxidase-conjugated secondary antibodies (anti-mouse IgG 1:5000, anti-rabbit IgG 1:5000, anti-goat IgG 1:5000) were obtained from Pierce Biotechnology, Inc. (Rockford, IL, USA).

### 3.15. Immunofluorescent Staining of AIF

At the indicated time after treatment, cells grown on cover slips were fixed with paraformaldehyde, permeabilized with 0.3% Triton X-100 and incubated with blocking solution containing 0.1% bovine serum albumin (BSA) in PBS. After washing with 8% BSA, cells were incubated overnight at 4 °C with primary antibody (1:100; rabbit polyclonal AIF, Santa Cruz Biotechnology, Inc.), diluted in 0.1% Triton X-100 in PBS (PBS-T) and then with secondary antibody (1:300; goat anti-rabbit IgG-FITC; Santa Cruz Biotechnology, Inc.). Cells were then washed with PBS and incubated at room temperature with DAPI (4′,6-diaminidino-2-phenyl-indole, dihydrochloride; 1 μg/mL) for nuclear visualization. After washing the cells with PBS, the coverslips were mounted on slides with MOWIOL and examined with fluorescence microscope (Leica DMI 6000 B, Leica Microsystems, Inc., Bannockburn, IL, USA).

### 3.16. Statistical Analysis

Data were processed with GraphPad Prism (GraphPad Software Inc., San Diego, CA, USA) and statistically analyzed using one-way ANOVA followed by Tukey’s multiple comparison test. All experimental groups were compared to control (^*^ *p* < 0.05, ^**^ *p* < 0.01, ^***^ *p* < 0.001), and the groups with combination treatment were compared to the groups treated with hypericin alone (^•^ *p* < 0.05, ^••^ *p* < 0.01, ^•••^ *p* < 0.001) or manumycin alone (^▪^ *p* < 0.05, ^▪▪^ *p* < 0.01, ^▪▪▪^ *p* < 0.001).

## 4. Conclusions

To sum up, in this study we report for the first time how the combination of photosensitive drug (hypericin) and farnesyltransferase inhibitor (manumycin) leads to increased antiproliferative and apoptotic response of adenocarcinoma cells HT-29 associated with reduced Ras protein levels. Enhanced apoptosis after combination treatment was demonstrated by elevated caspase-3/7 activity and time-dependent total cleavage of procaspase-3 and lamin B, cleavage of p21Bax into p18Bax and massive PARP cleavage. In addition, we discovered that Ras and AIF are new players in photoactivated hypericin action.
